# Validation of the rheumatoid arthritis diagnosis in the Swedish National patient register: a cohort study from Stockholm County

**DOI:** 10.1186/1471-2474-15-432

**Published:** 2014-12-15

**Authors:** Kristin Waldenlind, Jonas K Eriksson, Bernhard Grewin, Johan Askling

**Affiliations:** Department of Rheumatology, Karolinska University Hospital, Stockholm, Sweden; Clinical Epidemiology Unit, Department of Medicine Solna, Karolinska Institutet, SE-171 76 Stockholm, Sweden

**Keywords:** Epidemiology, Rheumatoid arthritis, Clinical registers, Validation

## Abstract

**Background:**

The Swedish National Patient Register offers unique possibilities for identification of large cohorts, such as patients with rheumatoid arthritis (RA). Although the overall diagnostic validity in the register has been reported as good, the aims of this study were to a) specifically validate the RA diagnosis from contemporary outpatient specialist care in this register, and b) assess the proportion of patients identified via algorithms to define incident RA in the register who in clinical practice also have new-onset disease.

**Methods:**

211 individuals with prevalent or incident RA in the National Patient Register were included. By extracting diagnosis-related parameters from their medical records, we determined if the patient fulfilled the 2010 ACR/EULAR- and the 1987 ACR-classification criteria for RA. We also determined whether clinical diagnosis was synchronous with disease onset as defined through register-based algorithms.

**Results:**

For 91% of the prevalent patients, the RA diagnosis in the National Patient Register fulfilled classification criteria or clinical diagnosis for RA. Among individuals identified with incident RA using a strict algorithm for new-onset disease, the RA diagnosis was substantiated in 91%, of whom 92% also represented new-onset disease.

**Conclusions:**

The validity of the RA diagnosis in the National Patient Register was high and, by using specific algorithms, new-onset RA can be defined. These findings strengthen the notion that the National Patient Register may be used to define RA populations with high validity to allow for high-quality epidemiological studies.

**Electronic supplementary material:**

The online version of this article (doi:10.1186/1471-2474-15-432) contains supplementary material, which is available to authorized users.

## Background

The extensive and high-quality Swedish health information network together with a population-based public health care system enables register-based epidemiological studies. For instance, studies on patients with rheumatoid arthritis (RA) have contributed to our understanding of the descriptive epidemiology of RA [1–5] and of several different aspects of the disease, its treatment and outcomes thereof [6–13]. In these studies the Swedish National Patient Register, maintained by The National Board of Health and Welfare, has commonly been used to identify patients with RA.

Hospital discharges from inpatient care and patients visits in non-primary outpatient care are registered in the National Patient Register. The register also includes the diagnosis in question, coded according to the International Classification of Diseases (ICD). The validity of the diagnosis codes for RA as assigned on discharge notes from hospitalization has previously been investigated [14]. However, only ad hoc and study-specific validations of diagnosis codes for RA from the outpatient component of the National Patient Register have been performed [15]. Since the vast majority of patients with RA are nowadays treated in outpatient care, it is important to assess the validity (or positive predictive value) of this component of the National Patient Register. In addition, register-based studies often seek to investigate morbidity in relation to onset of disease. Apart from validating the register-based RA diagnosis per se, it is also important to validate register-based algorithms to define incident RA.

The aims of this study were therefore to assess:

- the proportion of unselected (prevalent) patients listed with a RA-specific diagnosis from contemporary outpatient care in the National Patient Register who also fulfill formal classification criteria or a clinical diagnosis of RA
- the proportion of patients identified with incident RA who also have new-onset (incident) disease, using register-based algorithms.

To do this, we used the National Patient Register to identify prevalent and incident cases with RA. Chart abstraction was used to validate these register-based diagnoses against both classification criteria and clinical diagnoses. This is because classification criteria are not equal to diagnosis criteria.

## Methods

### Design and setting

The Swedish personal identification number is a 10-digit number unique to every Swedish resident. The National Patient Register, the Prescribed Drug Register and the medical files all contain the personal identification number, allowing for deterministic record linkage.

In Sweden, hospital-based rheumatologists treat the majority of all patients with RA. The Karolinska University Hospital in Stockholm houses the largest Rheumatology department in Sweden, providing around two thirds of all rheumatology care in the geographically defined Stockholm County Council (20% of the entire country).

### Data sources used

#### The national patient register

Hospital discharges from inpatient care have been registered since 1964 and patient visits in non-primary outpatient care since 2001. Diagnoses are coded according to the ICD. The coverage of inpatient discharges is close to 100% [16], whereas the coverage of outpatient discharges is around 87% [17]. The latter is higher for somatic care such as Rheumatology and for public care providers.

#### The prescribed drug register

The National Board of Health and Welfare maintain the Prescribed Drug Register. It contains information about all drugs dispensed on prescription in Sweden since 2005. The coverage is close to 100% (although in-hospital medication prescribed through non-electronic requisition is not included).

For this study, we identified two groups of patients from the National Patient Register, one with ‘prevalent’ RA and one with ‘incident’ RA. RA was defined as at least one listing with a primary or secondary ICD-10 diagnosis code of seropositive RA (M05.9) or seronegative RA (M06.0) between 2005 and 2008 at a non-primary outpatient care facility at the Karolinska University Hospital. Since the review period in the medical record was set to three years after being diagnosed with RA, the study period stretched from 2005 through 2012. In total, 211 patients were randomly extracted from the National Patient Register, equally allocated to a group of incident (according to an algorithm described below) and a group of prevalent RA.

Ethical approval was granted by the Regional Ethics Committee, Karolinska Institutet, Stockholm, Sweden. Reference number: DNR 2009/2005-31/3.

### Validation of the register-based diagnosis of RA in prevalent patients

The prevalent group consisted of 100 individuals defined as patients with two or more visits to a rheumatologist between 2005 and 2008, listing an ICD code for RA. The diagnosis was evaluated at the date of the second consecutive visit with RA according to the National Patient Register.

We developed a 20-item form to abstract diagnosis-related parameters [see Additional file 1]. By abstracting these parameters, it was possible to determine if the patient fulfilled the 2010 American College of Rheumatology (ACR)/European League Against Rheumatism (EULAR) classification criteria [18] and the 1987 ACR classification criteria [19] for RA. The criteria will be referred to as 2010 ACR/EULAR and 1987 ACR respectively in this text. Currently, both criteria are used in Swedish clinical practice to classify RA. In addition to the classification criteria, the following parameters were abstracted from the medical records:

1. Whether radiographic erosions had occurred either at a) the date the patient fulfilled the register based definition of prevalent RA (i.e. at the second visit listing this diagnosis), or at b) the end of the study period (three years later).
2. Whether the prevalent patient would clinically be regarded as having RA although not formally fulfilling the classification criteria at date of evaluation, or vice versa.

In those patients not fulfilling classification criteria or clinical diagnoses of RA, we also charted the alternative diagnoses in both the prevalent and incident group.

### Validation of the register-based diagnosis of RA in incident patients

The incident group consisted of 111 individuals defined as patients with their first ever visit to a rheumatologist in 2008, listing a RA diagnosis code using data from the National Patient Register. In addition to this *base-case* definition of incident RA, a *strict* definition of the register-based definition of incident RA was also explored. This definition required fulfillment of two additional criteria:

1. A second hospital visit for RA within one year after the first visit
2. No DMARD treatment more than 6 months before the first visit with RA diagnosis.

Information from the National Patient Register and the Prescribed Drug Register was used to identify patients with this strict algorithm.

Similar to the abstraction form used for evaluation of cases with a register-based definition of prevalent RA, we developed a 21-item form for patients with incident disease [see Additional file 2]. For the incident group the diagnosis was validated at ‘the incident time point’, the date of the first visits listing a diagnosis code for RA. We determined if the patient would clinically be regarded as having a new-onset (incident) disease, based on the medical history and the date of onset of RA symptoms. The abstraction forms were compiled manually. In addition to classification criteria, the following parameters were extracted from the medical records:

1. Whether radiographic erosions had occurred either at a) date of first visit, or b) two years after the first visit
2. Whether the RA diagnosis remained two years after the first visit
3. Date of onset of RA symptoms

## Results

The age and sex distribution of all patients compared to those with verified RA were similar, but with higher proportion of seropositive RA among the verified RA patients (Table 1).

**Table 1:** **Characteristics of the register-identified prevalent and incident RA patients**

	Prevalent RA		Incident RA		
	All patients	Verified RA	All patients	Verified RA	Verified incident RA
N	100	91	102	85	73
Women, n (%)	72 (72%)	64 (70%)	77 (75%)	65 (76%)	53 (73%)
Age					
- Mean (SD)	57.9 (16.1)	58.4 (16.1)	57.7 (17.4)	58.6 (17.3)	58.4 (17.8)
- Median (25^th^-75^th^)	58.4 (46.3-69.0)	59.1 (46.5-71.5)	60.0 (45.3-72.0)	60.7 (45.8-74.1)	60.7 (45.8-73.9)
RF positive, n (%)	85 (85%)	80 (88%)	70 (69%)	63 (74%)	53 (73%)

### Prevalent patients (n = 100)

Of the 100 patients identified as ‘prevalent RA’ in the National Patient Register, medical records for all were available for review. According to our register-based definition, chart review indicated that 91 (91%) were correctly diagnosed with RA. All but one of the remaining nine patients who did not have RA suffered from another inflammatory rheumatic disease (Table 2). Among the verified RA cases, 72 (79%) fulfilled both classification criteria for RA. In the seropositive subset (listed with an ICD-10 diagnosis code of seropositive/RF-positive RA), 80 out of 85 (94%) were correctly diagnosed with RA by at least one of the classification criteria. Of these, 50 (82%) fulfilled both classification criteria. Six patients did not fulfill the classification criteria at the date of evaluation. These all had a typical symmetric inflammatory polyarthritis and all the available information supported the diagnosis of RA. For these individuals, either of the classification criteria was generally fulfilled at a later stage.

**Table 2:** **Summary of medical records of** ***prevalent*** **patients registered with RA in the Swedish National Patient Register**

Total number of subjects	100
**Verified RA**	91(91%)
1987 ACR and 2010 ACR/EULAR criteria	72/91(79%)
1987 ACR criteria only	9/91(9.9%)
2010 ACR/EULAR criteria only	4/91(4.4%)
**RA diagnosis not substantiated**	9(9%)
*Granulomatosis with polyangiitis*	2
*Reactive arthritis*	1
*Polyarthritis*	4
*Unspecified arthralgia*	1
*Psoriatic arthritis*	1

n, values given as number of patients and (%) given as the equivalent percentage.

Forty-two (50%) of the patients with a verified RA-diagnosis had an erosive disease at the end of the observation period.

### Incident patients (n = 111)

Of the 111 patients identified with ‘incident RA’, 9 patients could not be validated because of an incomplete medical record with missing data. Hence they were excluded (Table 3).

**Table 3:** **Summary of medical records of** ***incident*** **patients in the** ***base-case definition*** **registered with RA in the Swedish National Patient Register**

Total number of subjects	111
Incomplete medical record	9
**Subjects amenable for validation**	102(100%)
**Verified RA**	85(83%)
**Thereof incident**	73(86%)
1987 ACR criteria and 2010 ACR/EULAR criteria	61/73(84%)
1987 ACR criteria only	9/73(12%)
2010 ACR/EULAR criteria only	1/73(1.4%)
**RA diagnosis not substantiated**	17(17%)
*Psoriatic arthritis*	6
*Juvenile ankylosing spondylitis*	1
*Unspecified polyarthritis*	5
*Borrelia arthritis*	1
*Systemic sclerosis*	1
*Systemic lupus erythematosus*	1
*Inflammatory systemic disease*	1
*Polymyalgia rheumatica*	1

n, values given as number of patients and (%) given as the equivalent percentage.

Chart review of the remaining patients indicated that 85 out of 102 patients (83%) were correctly coded with RA at date of first visit and remained so after the two-year follow-up period. Similar figures could be observed for the seropositive subgroup (63 out of 70; 90%). Of the patients whose RA diagnosis could not be substantiated, all suffered from another rheumatic disease according to their medical records (Table 3). Six patients fulfilled one or both of the classification criteria at date of first visit but not after the two-year follow-up period. For five of these cases, the initial RA diagnosis was changed to another rheumatic disease (Table 3).

Of the patients with verified RA diagnoses, 73 out of 85 (86%) also fulfilled our register-based base-case definition of ‘incident’ disease and did not develop any alternative explanation for their disease during the two years after first fulfillment of the RA diagnosis. The remaining patients were considered to be ‘prevalent’ since they all had a long-time history of RA, and had to a large extent been treated previously by a rheumatologist practicing outside the hospital. Among the verified incident RA cases 61 (84%) fulfilled both classification criteria for RA. Two of the incident RA cases fulfilled neither of the classification criteria at the date of evaluation, because information about the exact number of joints involved was lacking or the patient had palindromic rheumatism. However all the available information supported the diagnosis of RA. In some cases, there were missing data at the time point of evaluating the classification criteria, mainly about the existence of rheumatoid nodules and radiographic changes. Nevertheless they fulfilled the classification criteria and the missing data did not affect the outcome in these cases. Twenty-six patients (38%) of the verified incident RA cases had erosive disease at their first visit or developed erosive disease during the first two years after being diagnosed with RA. The median number of months between onsets of symptoms described in the medical file and recorded RA diagnosis in the National Patient Register was 6.7 months.

In the subgroup (n = 82) of patients identified as incident RA according to our ‘strict’ definition (Table 4), 75 (91%) were correctly diagnosed with RA. Of these, 69 (92%) were also incident.

**Table 4:** **Summary of medical records of** ***incident*** **patients in the** ***strict definition*** **of register-based incident RA in the Swedish National Patient Register**

Total number of subjects	82
**Verified RA**	75(91%)
**Thereof incident**	69(92%)
1987 ACR criteria and 2010 ACR/EULAR criteria	60/69(87%)
1987 criteria only	6/69(87%)
2010 ACR/EULAR criteria only	1/69(1.4%)
**RA diagnosis not substained**	7(8.5%)
*Psoriatic arthritis*	3
*Unspecified polyarthritis*	4

n, values given as number of patients and (%) given as the equivalent percentage.

## Discussion

The main findings of this study show that the validity of the register-based RA diagnosis was as high as 90% for prevalent RA and around 90% among cases identified, using strict algorithms to define incident RA.

There was no substantial difference in the validity when using the 1987 ACR or 2010 ACR/EULAR classification criteria, as the vast majority of patients fulfilled both. The number of patients classified with RA increased by approximately 10% when including patients only, fulfilling one of the two sets of classification criteria. The 2010 ACR/EULAR criteria were introduced to detect RA at an earlier phase than the 1987 ACR criteria [20]. However, somewhat counterintuitively, the majority of patients fulfilling only one of the two criteria in this study fulfilled the 1987 ACR criteria. These patients had mostly a seronegative RA with few joints involved. The patients only fulfilling the 2010 ACR/EULAR criteria were mostly patients with high positive anti-CCP or high positive RF and elevated ESR. Examining the medical records in some cases showed that the routine for documentation of joint involvement was less structured before the introduction of the 2010 ACR/EULAR criteria, which may have given rise to this result.

The validity was somewhat higher among patients receiving diagnosis codes for seropositive (vs. seronegative) disease. This is expected, as serological markers contribute to the classification criteria used as part of the ‘gold standard’.

Interestingly, we also noted that all patients but one who were incorrectly coded with RA suffered from another defined inflammatory rheumatic disease rather than unspecific arthralgia or osteoarthritis. These included Psoriatic Arthritis, Granulomatosis with polyangiitis (Wegener’s granulomatosis) and unspecified polyarthritis.

The prevalent patients with RA were required to have at least two visits to the department of rheumatology. Although the majority of the patients with prevalent RA had a long history of RA, a few of the prevalent patients (n = 9) had their two (first ever) visits close in time and were therefore also eligible as incident RA.

Since RA is a chronic disease and a long-lasting condition, the diagnosis can usually be verified over time. Some cases that fulfill the classification criteria for RA at one time point may at a later time point have developed another diagnosis than RA (e.g. Psoriatic Arthritis). A major strength of this study was the additional verification of the RA diagnosis for the incident patients through the follow-up visits approximately two years after the first visit. In this way it was possible to verify ‘genuine’ RA cases. However, using this gold standard may have resulted in an underestimation of the still good diagnostic accuracy. This is because the standard ensures that incident patients should not only have received a clinical diagnosis or fulfill the classification criteria at the ‘incident time point’ but they should also have retained their RA diagnosis after two years to be classified as having RA.

To our knowledge, previous validations of RA diagnoses in the outpatient component of the National Patient Register have been study-specific. Our present findings show a validity that correspond well to figures shown in one of the study-specific validations where over 90% of all diagnoses of an inflammatory poly-arthritide (RA M05 or M06, or other inflammatory poly-arthritis, M13) were correct [15]. Since the structure and coverage of health information networks and administrative databases differ between countries, our findings are not entirely comparable with similar international studies. However, the validity of an RA diagnosis among RA-coded individuals in the General Practice Research Database (database of primary care medical records in the UK-population) appears high for patients with specific characteristics. Using a data-derived diagnostic algorithm with a > 80% sensitivity and specificity, 61% of RA-coded patients fulfilled the diagnostic algorithm [21]. Another validation study using the medical records of rheumatologists in Ontario, Canada showed a high level of accuracy when using administrative data to identify RA patients. Most RA patients (84%) had an RA diagnosis present in the administrative data within +/- one year of a rheumatologist’s documented diagnosis date [22]. One retrospective chart abstraction study from the same area showed a positive predictive value ranging from 51-83% amongst the primary care sample, and ranging from 55-80% in the rheumatology sample, depending on the diagnostic algorithm used [23]. One study of discharge diagnoses for patients with RA recorded in the Danish National Patient Registry, showed an overall validity of 59%. However, major differences of the validity were seen depending on the characteristics of the underlying hospital registrations [24]. The design of our study allowed us to assess the positive predictive value of RA. In addition to the disease prevalence in the study sample the predictive values depend on the sensitivity and specificity of the diagnostic algorithm. Our study was not designed to assess sensitivity, specificity, nor the negative predictive value, however these could have been helpful validity measures. To this end, we note with interest that in previous studies of RA using data from the National Patient Register and from the Swedish Rheumatology Quality Register, the net contribution of patients from the latter was only 1%, suggesting a high coverage (‘sensitivity’) of the former.

One limitation of this study is the restricted number of patients. Although the Rheumatology department at the Karolinska University Hospital is the largest in Sweden, these results may not be generalized to the whole country. Nevertheless, validity data from this site is important, as it serves the vast majority of the country’s largest catchment area.

Besides the data sources used, the result of this study was based on the information given in the medical records. Therefore, the quality of the registration performed by the rheumatologist in the medical record significantly impacted the outcome. For example, a structured documentation of joint involvement was a prerequisite for validation of the classification criteria. This is why a few patients (N = 9) were excluded since the medical record was incomplete.

## Conclusions

Based on medical records from patients coded with RA in the National Patient Register in Stockholm County, the validity of the RA diagnosis was high both in prevalent patients and for patients identified with new-onset RA. These findings strengthen the notion that the National Patient Register and data algorithms may be used to define RA populations with a sufficiently high validity to allow for high-quality epidemiological studies.

## Electronic supplementary material

Additional file 1:Abstraction form for validation of Rheumatoid Arthritis diagnosis in the Swedish National Patient Register: Prevalent patients.(PDF 617 KB)

Additional file 2:Abstraction form for validation of Rheumatoid Arthritis diagnosis in the Swedish National Patient Register: Incident patients.(PDF 618 KB)

## Abbreviations

RA: Rheumatoid arthritis
ACR: American College of Rheumatology
EULAR: European League Against Rheumatism
ICD: International Classification of Diseases.

## References

[1] Askling J, Fored CM, Geborek P, Jacobsson LT, van Vollenhoven R, Feltelius N, Lindblad S, Klareskog L (2006). Swedish registers to examine drug safety and clinical issues in RA. Ann Rheum Dis.

[2] Eriksson JK, Neovius M, Ernestam S, Lindblad S, Simard JF, Askling J (2013). Incidence of rheumatoid arthritis in Sweden: a nationwide population-based assessment of incidence, its determinants, and treatment penetration. Arthritis Care Res.

[3] Neovius M, Simard J, Sundstrom A, Jacobsson L, Geborek P, Saxne T, Feltelius N, Klareskog L, Askling J, Group AS (2011). Generalisability of clinical registers used for drug safety and comparative effectiveness research: coverage of the Swedish Biologics Register. Ann Rheum Dis.

[4] Neovius M, Simard JF, Askling J (2011). group As: Nationwide prevalence of rheumatoid arthritis and penetration of disease-modifying drugs in Sweden. Ann Rheum Dis.

[5] Neovius M, Sundstrom A, Simard J, Wettermark B, Cars T, Feltelius N, Askling J, Klareskog L, Group AS (2011). Small-area variations in sales of TNF inhibitors in Sweden between 2000 and 2009. Scand J Rheumatol.

[6] Askling J, Baecklund E, Granath F, Geborek P, Fored M, Backlin C, Bertilsson L, Coster L, Jacobsson LT, Lindblad S, Lysholm J, Rantapää-Dahlqvist S, Saxne T, van Vollenhoven R, Klareskog L, Feltelius N (2009). Anti-tumour necrosis factor therapy in rheumatoid arthritis and risk of malignant lymphomas: relative risks and time trends in the Swedish Biologics Register. Ann Rheum Dis.

[7] Askling J, Dixon W (2008). The safety of anti-tumour necrosis factor therapy in rheumatoid arthritis. Curr Opin Rheumatol.

[8] Askling J, Dixon W (2011). Influence of biological agents on cardiovascular disease in rheumatoid arthritis. Ann Rheum Dis.

[9] Askling J, Fored CM, Brandt L, Baecklund E, Bertilsson L, Feltelius N, Coster L, Geborek P, Jacobsson LT, Lindblad S, Lysholm J, Rantapää-Dahlqvist S, Saxne T, van Vollenhoven RF, Klareskog L (2007). Time-dependent increase in risk of hospitalisation with infection among Swedish RA patients treated with TNF antagonists. Ann Rheum Dis.

[10] Askling J, van Vollenhoven RF, Granath F, Raaschou P, Fored CM, Baecklund E, Dackhammar C, Feltelius N, Coster L, Geborek P, Jacobsson LT, Lindblad S, Rantapää-Dahlqvist S, Saxne T, Klareskog L (2009). Cancer risk in patients with rheumatoid arthritis treated with anti-tumor necrosis factor alpha therapies: does the risk change with the time since start of treatment?. Arthritis Rheum.

[11] Raaschou P, Simard JF, Holmqvist M, Askling J, Group AS (2013). Rheumatoid arthritis, anti-tumour necrosis factor therapy, and risk of malignant melanoma: nationwide population based prospective cohort study from Sweden. BMJ.

[12] Raaschou P, Simard JF, Neovius M, Askling J, Anti-Rheumatic Therapy in Sweden Study G (2011). Does cancer that occurs during or after anti-tumor necrosis factor therapy have a worse prognosis? A national assessment of overall and site-specific cancer survival in rheumatoid arthritis patients treated with biologic agents. Arthritis Rheum.

[13] Simard JF, Neovius M, Askling J, Group AS (2012). Mortality rates in patients with rheumatoid arthritis treated with tumor necrosis factor inhibitors: drug-specific comparisons in the Swedish Biologics Register. Arthritis Rheum.

[14] Baecklund E, Iliadou A, Askling J, Ekbom A, Backlin C, Granath F, Catrina AI, Rosenquist R, Feltelius N, Sundstrom C, Klareskog L (2006). Association of chronic inflammation, not its treatment, with increased lymphoma risk in rheumatoid arthritis. Arthritis Rheum.

[15] Knight A, Sandin S, Askling J (2010). Increased risk of autoimmune disease in families with Wegener's granulomatosis. J Rheumatol.

[16] Ludvigsson JF, Andersson E, Ekbom A, Feychting M, Kim JL, Reuterwall C, Heurgren M, Olausson PO (2011). External review and validation of the Swedish national inpatient register. BMC Public Health.

[17] Socialstyrelsen (2013). Kodningskvalitet i patientregistret – Ett nytt verktyg för att mäta kvalitet.

[18] Aletaha D, Neogi T, Silman AJ, Funovits J, Felson DT, Bingham CO, Birnbaum NS, Burmester GR, Bykerk VP, Cohen MD, Combe B, Costenbader KH, Dougados M, Emery P, Ferraccioli G, Hazes JM, Hobbs K, Huizinga TW, Kavanaugh A, Kay J, Kvien TK, Laing T, Mease P, Ménard HA, Moreland LW, Naden RL, Pincus T, Smolen JS, Stanislawska-Biernat E, Symmons D (2010). 2010 Rheumatoid arthritis classification criteria: an American College of Rheumatology/European League Against Rheumatism collaborative initiative. Arthritis Rheum.

[19] Arnett FC, Edworthy SM, Bloch DA, McShane DJ, Fries JF, Cooper NS, Healey LA, Kaplan SR, Liang MH, Luthra HS, Medsger TA, Mitchell DM, Neustadt DH, Pinals RS, Schaller JG, Sharp JT, Wilder RL, Hunder GG (1988). The American Rheumatism Association 1987 revised criteria for the classification of rheumatoid arthritis. Arthritis Rheum.

[20] Biliavska I, Stamm TA, Martinez-Avila J, Huizinga TW, Landewe RB, Steiner G, Aletaha D, Smolen JS, Machold KP (2013). Application of the 2010 ACR/EULAR classification criteria in patients with very early inflammatory arthritis: analysis of sensitivity, specificity and predictive values in the SAVE study cohort. Ann Rheum Dis.

[21] Thomas SL, Edwards CJ, Smeeth L, Cooper C, Hall AJ (2008). How accurate are diagnoses for rheumatoid arthritis and juvenile idiopathic arthritis in the general practice research database?. Arthritis Rheum.

[22] Widdifield J, Bernatsky S, Paterson JM, Tu K, Ng R, Thorne JC, Pope JE, Bombardier C (2013). Accuracy of Canadian health administrative databases in identifying patients with rheumatoid arthritis: a validation study using the medical records of rheumatologists. Arthritis Care Res.

[23] Widdifield J, Bombardier C, Bernatsky S, Paterson JM, Green D, Young J, Ivers N, Butt DA, Jaakkimainen RL, Thorne JC, Tu K (2014). An administrative data validation study of the accuracy of algorithms for identifying rheumatoid arthritis: the influence of the reference standard on algorithm performance. BMC Musculoskelet Disord.

[24] Pedersen M, Klarlund M, Jacobsen S, Svendsen AJ, Frisch M (2004). Validity of rheumatoid arthritis diagnoses in the Danish National Patient Registry. Eur J Epidemiol.

[CR25] The pre-publication history for this paper can be accessed here:http://www.biomedcentral.com/1471-2474/15/432/prepub

