# Supplementary material for: Validation of the rheumatoid arthritis diagnosis in the Swedish National patient register: a cohort study from Stockholm County
Source: BMC Musculoskelet Disord. 2014 Dec 15;15:432. doi: 10.1186/1471-2474-15-432 (PMC4302140; doi:10.1186/1471-2474-15-432)
Supplement: Supplementary file 1 — Additional file 1:Abstraction form for validation of Rheumatoid Arthritis diagnosis in the Swedish National Patient Register: Prevalent patients.(PDF 617 KB) [file 12891_2014_2378_MOESM1_ESM.pdf]

Additional file 1

Form for validation of Rheumatoid Arthritis diagnosis in the Swedish national patient register and the Swedish Rheumatology Quality register (SRQ)

Prevalent patients: two visits at a rheumatologist listing an RA-diagnosis between 2005-2008

The 1987 ACR classification criteria for rheumatoid arthritis

4 out of 7 criteria with symptoms at least 6 weeks is needed for classification of a patient as having RA

	yes	no	missing data
**1)** Morning stiffness- at least 1 hour			
**2**) Arthritis in at least 3 out of 14 joint areas(pip-, mcp-, wrist-, elbow-, knee-, ankle-, mtp-joints)			
**3)** Arthritis of hand joints(mcp-,pip-,wrists)			
**4)** Symmetric arthritis (mcp-, pip-, mtp-joints)			
**5)** Rheumatoid nodules			
**6)** Positive Serum Rheumatoid factor			
**7)** Radiographic changes (typical of rheumatoid arthritis on posteroanterior hand and wrist radiographs)			
**8**) Fullfills criteria			

The 2010 ACR-EULAR classification criteria för rheumatoid arthritis

Patient with at least one joint with definitive clinical synovitis .=/>6 points is needed for classification of a patient as having definite RA

	Points	
**A. Joint involvment-** Refers to any swollen or tender joint on examination		
DIP, CMC 1 and MTP- 1 joints excluded from assessment		
1 large joint (shoulder, elbow, hip, knee, ankle)	0	
2-10 large joints	1	
1-3 small joint (mcp, pip, mtp 2-5, wrist, ip-1)-with or without involvment of large joints	2	
4-10 small joints- with or without involvment of large joints	3	
> 10 joints- at least one small joint(also including other joints ie. AC, SC)	5	
**B.** Serology(at least 1 test result is needed for classification)		
Negative RF *and* negative ACPA	0	
Low positive RF *or* low positive ACPA	2	
High positive RF *or* high positive ACPA	3	
Low positive: IU values > the ULN but < 3 times the ULN .		
High positive: > 3 times the ULN. If RF information only available as positiv=low positive		
**C.** Acute-phase reactants		
Normal CRP *and* normal ESR	0	
Abnormal CRP *or* abnormal ESR	1	
**D.** Duration of symptoms(patient self-report of the duration of signs or symptoms of synovitis)		
<6 weeks	0	
>6 weeks	1	
**E.** Fulfills criteria		

<u>Additional information</u>

RA-diagnosis in the NPR(date when the RA-diagnosis was validated)	date			
Included in the SRQ	date			
Erosive disease when listed as prevalent RA (based on hand and feet radiographs)				
Erosive disease 3 years after listed as prevalent RA(based on hand and feet radiographs)				
Clinical RA				
Other diagnosis if not RA/co-morbidity/comment(based on the medical record in Take Care)				
