# Supplementary material for: Validation of the rheumatoid arthritis diagnosis in the Swedish National patient register: a cohort study from Stockholm County
Source: BMC Musculoskelet Disord. 2014 Dec 15;15:432. doi: 10.1186/1471-2474-15-432 (PMC4302140; doi:10.1186/1471-2474-15-432)
Supplement: Supplementary file 2 — Additional file 2:Abstraction form for validation of Rheumatoid Arthritis diagnosis in the Swedish National Patient Register: Incident patients.(PDF 618 KB) [file 12891_2014_2378_MOESM2_ESM.pdf]

Additional file 2

Form for validation of Rheumatoid Arthritis diagnosis in the Swedish national patient register and the Swedish Rheumatology Quality register(SRQ)

Incident patients: diagnosed with RA in 2008

The 1987 ACR classification criteria for rheumatoid arthritis

4 out of 7 criteria with symptoms at least 6 weeks is needed for classification of a patient as having RA

		yes	no	missing data
**1)** Morning stiffness- at least 1 hour				
**2**) Arthritis in at least 3 out of 14 joint areas(pip-, mcp-, wrist-, elbow-, knee-, ankle-, mtp-joints)				
**3)** Arthritis of hand joints(mcp-,pip-,wrists)				
**4)** Symmetric arthritis (mcp-, pip-, mtp-joints)				
**5)** Rheumatoid nodules				
**6)** Positive Serum Rheumatoid factor				
**7)** Radiographic changes (typical of rheumatoid arthritis on posteroanterior hand and wrist radiographs)				
**8**) Fullfills criteria				

The 2010 ACR-EULAR classification criteria för rheumatoid arthritis

Patient with at least one joint with definitive clinical synovitis .=/>6 points is needed for classification of a patient as having definite RA

		Points	
**A. Joint involvment-** Refers to any swollen or tender joint on examination			
DIP, CMC 1 and MTP- 1 joints excluded from assessment			
1 large joint (shoulder, elbow, hip, knee, ankle)		0	
2-10 large joints		1	
1-3 small joint (mcp, pip, mtp 2-5, wrist, ip-1)-with or without involvment of large joints		2	
4-10 small joints- with or without involvment of large joints		3	
> 10 joints- at least one small joint(also including other joints ie. AC, SC)		5	
**B.** Serology(at least 1 test result is needed for classification)			
Negative RF *and* negative ACPA		0	
Low positive RF *or* low positive ACPA		2	
High positive RF *or* high positive ACPA		3	
Low positive: IU values > the ULN but < 3 times the ULN .			
High positive: > 3 times the ULN. If RF information only available as positiv=low positive			
**C.** Acute-phase reactants			
Normal CRP *and* normal ESR		0	
Abnormal CRP *or* abnormal ESR		1	
**D.** Duration of symptoms(patient self-report of the duration of signs or symptoms of synovitis)			
<6 weeks		0	
>6 weeks		1	
**E.** Fulfills criteria			

Additional information

RA-diagnosis in the NPR (date when the RA-diagnosis was validated)	date			
Included in the SRQ	date			
Clinical RA diagnosis (based on the medical journal in take care)	date			
Date of onset of symptoms (based on the medical journal in take care and SRQ)				
Erosive disease 1 year after RA-diagnosis (based on hand and feet radiographs)				
Erosive disease 2 years after RA-diagnosis(based on hand and feet radiographs)				
Remaining RA diagnosis after 2 years (based on the medical record in take care)				
Other diagnosis if not RA/co-morbidity/comment(based on the medical record in Take Care)				
